# Secondary bacterial infections & extensively drug-resistant bacteria among COVID-19 hospitalized patients at the University Hospital in Kraków

**DOI:** 10.1186/s12941-023-00625-8

**Published:** 2023-08-24

**Authors:** A Pałka, A Kujawska, DA Hareza, M Gajda, Jerzy Wordliczek, E Jachowicz-Matczak, I Owsianka, B Żółtowska, A Chmielarczyk, D Romaniszyn, Gregorczyk-Maga I, J Wójkowska-Mach

**Affiliations:** 1 Department of Microbiology, University Hospital in Krakow, ul. Jakubowskiego 2, 30-688 Kraków, Poland; 2 Division of Infectious Diseases, Johns Hopkins School of Medicine, 1830 E Monument St, 4th Floor, Baltimore, MD USA; 3 Chair of Microbiology, Faculty of Medicine, Jagiellonian University Medical College, ul. Czysta 18, 31-121 Kraków, Poland; 4 Doctoral School of Medicine and Health Sciences, Jagiellonian University Medical College, ul. św. Anny 12, 31- 008 Kraków, Poland; 5 Anesthesiology and Intensive Care Clinical Department, University Hospital in Krakow, ul. Jakubowskiego 2, 30- 688 Kraków, Poland; 6 Institute of Dentistry, Faculty of Medicine, Jagiellonian University Medical College, ul. Montelupich 4, 31-155 Kraków, Poland

**Keywords:** Extensively drug-resistant, HAI, Bloodstream infections (BSI), Intensive care unit (ICU), Poland, *Acinetobacter baumannii*

## Abstract

**Introduction:**

Healthcare-associated infections (HAI) and bacterial antimicrobial resistance posed a therapeutic risk during the coronavirus disease 2019 (COVID-19) pandemic. The aim of this study was to analyze the HAIs in COVID-19 patients in the Intensive Care Unit (ICU) and non-ICU at the University Hospital in Krakow (UHK) with an emphasis on the susceptibility of the most frequently isolated pathogens and the prevalence of extensively drug resistant (XDR) microorganisms.

**Methods:**

This laboratory-based study was carried out at the University Hospital in Krakow in the ICU and non-ICUs dedicated to COVID-19 patients between May 2021 and January 2022. All isolates of *Klebsiella pneumoniae* were analyzed using PFGE protocol.

**Results:**

292 independent HAI cases were identified, with the predominance of urinary tract infections (UTI), especially in the non-ICU setting. The most common ICU syndrome was pneumonia (PNA). The prevalence of XDR organisms was 22.6% in the ICU and 14.8% in non-ICUs among all isolates. The incidence of carbapenem-resistant Enterobacteriaceae infection was 24.8 cases per 10,000 hospitalizations and the carbapenem-resistant *A. baumannii* infection incidence was 208.8 cases per 10,000 hospitalizations. The prevalence of XDR strains was highest in *Acinetobacter spp*, in PNA cases. The PFGE typing demonstrated that almost all XDR strains varied widely from each other.

**Conclusions:**

In this study, there was a high incidence of HAI in COVID-19 patients, especially when compared to Western Europe and the United States. Similarly, the prevalence of XDR microorganisms, especially XDR-*A.baumannii*, was also high. PFGE did not confirm the horizontal spread of any organism strains.

**Supplementary Information:**

The online version contains supplementary material available at 10.1186/s12941-023-00625-8.

## Introduction

Bacterial antimicrobial resistance (AMR) has emerged as one of the leading public health threats of the 21st century; researchers agree that the spread of AMR is an urgent issue requiring a coordinated, global action plan to address. The problem particularly concerns the most important groups of antibiotics, the beta-lactams (antibiotics considered first-line for empiric therapy of severe infections) and fluoroquinolones, because resistance to them accounted for more than 70% of global deaths attributable to bacterial AMR in 2019 [1]. However, not all AMR poses the same therapeutic problem, and epidemiological surveillance is even more relevant for multi (MDR) and extensively drug-resistant (XDR) pathogens –MDR defined as acquired non-susceptibility to at least one agent in three or more antimicrobial categories and XDR defined as non-susceptibility to at least one agent in all but two or fewer antimicrobial categories – that cause severe and often fatal infections in healthcare settings and nursing homes. The prevalence of XDR bacteria can be hard to estimate; the data are scarce. In one study conducted at a tertiary care centre in central India, 13.8% of bacterial strains were XDR [2] but this can vary widely based on location. In addition to an XDR-infection, factors that impact patient mortality include a critical illness, especially requiring a stay in the Intensive Care Unit (ICU), extensive comorbidities, and consecutive episodes of septic shock [3].

AMR is a diverse threat, and its effects vary depending on the pathogen. One of the most concerning is *Acinetobacter baumannii*. According to a systematic review by Xie et al., its level of resistance to carbapenems is extremely geographically variable. The pooled prevalence in hospital settings ranges from 54% (95% CI 36.8–70.8) in OECD (Organization for Economic Co-operation and Development) countries to 77% (95% CI 67.4–86.2) in non-OECD countries [4].* A. baumannii* XDR strains are common due to a diverse and extensive arsenal of chromosomally encoded and/or acquired resistance genes [5]. *Pseudomonas aeruginosa* resistance to carbapenems is also genetically diverse and usually exhibits more than one resistance mechanism [6]. Enterobacterales (mainly *Klebsiella pneumoniae*, but also *Enterobacter cloacae*, *Escherichia coli*, *Citrobacter* spp. and *Serratia marcescens*) resistance to carbapenems is due to the production of a wide variety of β-lactamases, which often confer resistance to almost all β-lactam antibiotics. MBL (metallo-β-lactamase) production, often of the NDM type (New Delhi metallo-β-lactamase), ranges from 83%, 13%, 1%, and 1% in Asia, Europe, USA and Africa, respectively [7]. In turn, KPC (*Klebsiella pneumoniae* carbapenemase) class A carbapenemases are more widely spread in the Americas, Korea and China [8]. The prevalence of resistance to carbapenems varies widely across Europe, with the highest rates in the Mediterranean and Balkan countries [9]. It is estimated that 33,000 patients die every year in Europe due to infections caused by antibiotic-resistant bacteria [10], which include carbapenem-resistant microorganisms, due to a lack of effective and safe alternative treatment options.

As a consequence of the high incidence and morbidity of COVID-19 (coronavirus disease 2019), there was a dramatic increase in the number of hospitalized ICU patients in Poland from 2020 to 2021 [11]. To accommodate the influx of patients, hospitals were organized into dedicated units for patients infected with SARS-CoV-2 (COVID-19 units). Due to their isolation, these specialized units should have reduced the risk of microbial transmission, including XDR bacteria. However, these units experienced overload due to the rapid increase in patient admissions which also resulted in a shortage of personal protective equipment (PPE) that risked transmission of microbes [12]. In addition, a concern is that the increased use of antibiotics during the pandemic to treat patients with COVID-19 may have facilitated the spread of resistant bacteria. Some patients admitted to hospitals received empiric antibacterial therapy, which was not always indicated, thus potentially increasing the risk for selection of resistant bacteria [13].

The aim of this study was to analyze the type of HAIs in patients in temporary covid units (the ICU and non-ICU) at the University Hospital in Krakow (UHK). At the same time, an attempt was made to determine the species profile of these infections and the susceptibility to antibiotics of the most frequently isolated pathogens.

## Methods

### Setting

The study was carried out at the University Hospital in Krakow (UHK), the largest teaching hospital in Southern Poland. It has 39 clinical departments (1310 beds in total), 2 intensive care departments (40 beds), 7 institutes, and 68 outpatient clinics. In the beginning of the COVID-19 pandemic (March 2020 through September 2020), UHK was devoted solely to treating COVID-19 patients. From October 2020 to March 2022, these patients were hospitalized in a separate building where 200 adult beds, including 50 intensive care beds, were created. Each patient admitted to the hospital was tested for SARS-CoV-2 virus by PCR (COBAS 6800, Roche or in the CITO mode GeneXpert System, Cepheid, USA) regardless of chief complaint or symptoms. 131 doctors and 433 nurses staffed these COVID-19 units.

This is a laboratory-based study; the samples were collected between May 1st, 2021, and January 31st, 2022. A bacterial healthcare-associated infection (HAI) was defined as a symptomatic infection in which the positive culture was taken a minimum of 48 h since hospital admission. HAI cases (not including *C. difficile* infections) were analyzed retrospectively using definitions from the Healthcare-Associated Infections Surveillance Network (HAI-Net, https://ecdc.europa.eu/sites/portal/files/documents/HAI-Net-ICU-protocol-v2.2_0.pdf.), including bloodstream infections (BSI), pneumonias (PNA), urinary tract infections (UTI) and others. The samples were obtained via passive surveillance wherein collection of samples relied upon healthcare personnel taking the initiative to identify and report HAIs based on reporting guidelines.

### Bacterial isolates

Microbial samples were taken from the sites of infection. Only laboratory confirmed HAIs cases based on culture growth qualified for the analysis; only the first isolate from each HAI case was analyzed as well. An HAI case was defined as a specific infectious syndrome (i.e., PNA) from a particular organism. If another syndrome (i.e., UTI) occurred at least 3 days later with a different organism, it would be counted as a separate HAI case and both organisms would be analyzed. In total, 327 clinical strains qualified for the study. Colonizing strains were not included in the study and were not analyzed.

Bacterial strains were identified by mass spectrometry (VITEK MS, Biomerieux, France). Drug susceptibility was performed by the hospital diagnostic laboratory using the automatic VITEK 2 method (Biomerieux, France), disc diffusion (OXOID, UK), the E-test method (LIOFILCHEM, Italy) or the broth microdilution method in the case of colistin (MIC STRIPPED PLATES COL; Diagnostics, Slovakia), according to the current guidelines of the European Committee on Antimicrobial Susceptibility Testing (EUCAST https://www.eucast.org/clinical_breakpoints/). Determination of resistance mechanisms was performed using the disk diffusion method (OXOID, UK) and the E-test method (LIOFILCHEM, Italy). When checking for carbapenemases (types: VIM, NDM, IMP, KPC, OXA-48), the NG-Test Carba 5 cassette test (NG-Biotech, France) and the PCR method (GeneXpert System, Cepheid, USA) were used.

The disc diffusion method was used for the detection of the following isolates: MRSA (methicillin resistant *Staphylococcus aureus*) with a 30 µg cefoxitin disc; VRE (vancomycin-resistant *Enterococcus*) with a 5 µg vancomycin disc; CPE (carbapenemase-producing *Enterobacteriaceae)* and KPC (*Klebsiella pneumoniae* carbapenemase-producing bacteria) using phenylboronic acid (15 µg / ml), 10 µg of meropenem and 10 µg of imipenem; MBL (metallo-β-lactamase producing bacteria) with acid EDTA (Ethylenediaminetetraacetic acid), 10 µg of imipenem and 30 µg of ceftazidime; and OXA beta-lactamases using *OXA-48* discs with a 30 µg temocillin disc. The E-test method was also used for detecting the mechanism of VRE.

### Pulsed-field gel electrophoresis (PFGE)

All isolates of *K. pneumoniae* were analyzed using the standardized PFGE protocol developed at the Centers for Disease Control and Prevention (CDC) by the PulseNet program (version for *E. coli*). Due to previous studies [14] showing the possibility of clonal spread of certain *K. pneumoniae* strains in Polish hospitals, a PFGE study was performed for this species. Genomic DNA was prepared in situ in agarose blocks and was subsequently digested with restriction enzymes: XbaI (25U per block, Thermo Scientific). The digested products were separated on a CHEF III PFGE system (BioRad, Warsaw, Poland) in 0.5 × Tris-borate-EDTA buffer at 14 °C at 6 V for 22 h with a starting pulse of 2s and final pulse of 35s. GelCompar (Applied Maths, Kortrijk, Belgium) was used for cluster analysis with the unweighted pair group method with an arithmetic mean and the Dice coefficient. The similarity requirement for the pattern to be considered as belonging to the same type was > 90% (https://www.cdc.gov/pulsenet/pathogens/pfge.html).

### Statistical analysis

In the statistical analysis of the data, relative and absolute frequencies were used for nominal variables and the median with quartiles (Q1, Q3) for quantitative variables (age). Chi2 test and Student’s t-test were used to compare the groups of patients with XDR vs. without XDR, sex, sample material, type of bacteria and the Mann–Whitney U test was used for the age variable. The risk of XDR was assessed in a multivariable logistic regression model. The analysis was carried out in SPSS ver. 26. In all analyses, the significance level was α = 0.05.

### Ethics approval and consent to participate

This work was approved by the Bioethics Committee of the Jagiellonian University (approval no. 1072.6120.353.2020 from 16.12.2020). All data analyzed during this study was anonymized prior to analysis.

## Results

During the study period, between May 1st, 2021 and January 31st, 2022, there was a total of 2,826 patients with COVID-19 in the temporary COVID-19 units, the majority (57%) of whom were hospitalized in the ICU. The median length of stay was significantly dependent on the type of ward; it was 11 days in the ICU (interquartile range (IQR) 7–15) and 14 days in the non-ICU setting (IQR 7–22, Table 1). Altogether, 292 independent HAI cases were identified with the predominance of UTIs (121 cases, 41% of all HAIs), especially in the non-ICU setting (48 cases, 69.9% of all HAIs in the non-ICU, Table 2). The most common ICU syndrome was PNA with 103 cases (46.2% of all HAIs in the ICU).

**Table 1:** Basic patient characteristics and hospitalization data in the temporary COVID-19 units of the University Hospital in Krakow

Demographics	ICU		Non-ICU		p-value
Sex					
Female	733	45.4%	418	34.5%	< 0.001
Male	881	54.6%	794	65.5%	
**Age [years]**					
< 65	616	38.2%	507	41.8%	< 0.001
65–74	446	27.6%	537	44.3%	
>=75	552	34.2%	168	13.9%	
Length of stay, median (Q1;Q3) [days]	11.0 [7.0;15.0]		14.0 [7.0;22.0]		< 0.001*
Admissions, [n]	1 614		1 212		
Patient-days of stay [days]	18 810		22 629		
Patients with hospitalization length ≥ 3 days, [n]	1 486		1 125		
Patient-days of stay hospitalizations ≥ 3 [days]	18 670		22 504		
Number of bacterial HAIs	223		69		

Legend: HAI: healthcare-associated infections; ICU: intensive care unit; Q1: first quartile; Q3: third quartile

*Mann-Whitney U test

**Table 2:** Bacterial HAIs in ICU and non-ICU settings at UHK

Bacterial HAIs	ICU		Non-ICU	
	N	%	N	%
PNA	103	46.2	7	10.1
UTI	73	33.0	48	69.9
BSI	41	18.8	9	13.2
Others	6	3.8	5	7.2
**Total**	**223**	100.0	**69**	100.0
Bacterial HAI, incidence %	13.8%		5.7%	
Bacterial HAI, incidence, per 1000pds	11.9		3.0	
XDR prevalence**	**%**		**%**	
PNA	39	27.5	1	12.5
UTI	19	20.7	8	14.3
BSI	6	12.8	1	10.0
Others	1	14.3	2	28.0
**Total**	**65**	**22.6**	**12**	**14.8**
**Prevalence of specific resistance mechanisms**	**%**		**%**	
MRSA, N = 6	13%		40%	
VRE, N = 16	26%		25%	
KPC, N = 5	10%		14%	
OXA-48, N = 1	2%		0.0%	
MBL*, N = 0	0.0%		0.0%	
CR-*A. baumannii*, N = 52	93%		0.0%	
**Incidence rate of specific resistance mechanisms**	**per 10,000 hospitalizations**		**per 10,000 hospitalizations**	
CR Enterobacteriaceae	37.2		8.3	
CR *A. baumannii*	359.7		8.3	

Legend: BSI: bloodstream infection; CR: carbapenem resistance; HAI: healthcare-associated infections; ICU: intensive care unit; KPC: *Klebsiella pneumoniae* carbapenemase (KPC)-producing bacteria; MBL: metallo-β-lactamase; MRSA: methicillin-resistant *Staphylococcus aureus*; OXA-48: OXA-48-type carbapenemases; pds: patient days; PNA: pneumonia; UTI: urinary tract infection; VRE: Vancomycin-resistant *Enterococcus*; XDR: extensively drug-resistant; UHK: University Hospital in Krakow

*Evaluated only for *E. coli, K. pneumoniae*

**Calculated as: (No of XDR-HAI/all HAIs) *100

The HAI incidence was 13.8% in the ICU and 5.7% in non-ICUs; the incidence density was 11.9 and 3.0 per 1000 patient days (pds) (Table 2), respectively. The prevalence of XDR organisms was 22.6% in the ICU and 14.8% in non-ICUs among all isolates. The most common HAIs associated with XDR organisms was PNA, both in the ICU and non-ICUs, at 27.5% and 12.5%, respectively (Table 2).

The prevalence of XDR strains was highest in *Acinetobacter spp* (81.5% of all *Acinetobacter spp* isolates), in PNA cases (33.9% of PNA cases) and in males (28.3% of males) (Table 3). The factor that significantly increased the prevalence of XDR was infection with *A. baumannii* (OR 46.8, 95% CI 21.27; 103.09, p 0.001) (Table 4). XDR prevalence was significantly lower in women than in men (OR 0.4, 95% CI 0.20–0.99, p 0.001, Table 4).

**Table 3:** Extensively drug-resistant strain prevalence in COVID-19 patients at UHK

XDR prevalence according to selected factors [%]	XDR		p-value
	No	Yes	
**Age median (Q1;Q3) [years]**	69 (63;75)	66 (63;73)	0.106
**HAIs N** [%]			
PNA	78 (66.1%)	40 (33.9%)	0.008
UTI	107 (79.9%)	27 (20.1%)	
BSI	48 (87.3%)	7 (12.7%)	
Others	14 (82.4%)	3 (17.6%)	
**ICU N** [%]			
Yes	196 (75.1%)	65 (24.9%)	0.415
No	51 (81.0%)	12 (19.0%)	
**Sex N** [%]			
Female	103 (83.1%)	21 (16.9%)	0.029
Male	142 (71.7%)	56 (28.3%)	
**Etiological factors** N [%]			
* Staphylococcus aureus*	36 (100.0%)	0 (0.0%)	< 0.001
* Enterococcus spp.*	60 (100.0%)	0 (0.0%)	
* Escherichia coli*	45 (100.0%)	0 (0.0%)	
* Klebsiella spp.*	32 (75.0%)	11 (25.0%)	
* Acinetobacter spp*	12 (19.0%)	51 (81.0%)	
Others	55 (80.9%)	13 (19.1%)	

Legend: BSI: bloodstream infection; HAI: healthcare-associated infections; ICU: intensive care unit; PNA: pneumonia; UTI: urinary tract infection; Q1: first quartile; Q3: third quartile; XDR: extensively drug-resistant; UHK: University Hospital in Kraków

**Table 4:** Multivariate analysis of risk factors for extensively drug-resistant (XDR) strains

	OR	95% CI	p-value
ICU vs. non-ICU	0.5	(0.22; 1.10)	0.086
Sex	0.4	(0.20; 0.99)	0.048
Age	0.9	(0.97; 0.99)	< 0.001
*Acinetobacter* Yes vs. No	46.8	(21.27; 103.09)	< 0.001

Nagelkerke R2 = 0.680

Legend: ICU: intensive care unit; OR: odds ratio; 95% CI: 95% confidence interval

Carbapenem-resistant *Enterobacteriaceae* infection incidence was 24.8 cases per 10,000 hospitalizations (37.2 for the ICU and 8.3 for non-ICUs) and carbapenem-resistant *A. baumannii* infection incidence was 208.8 cases per 10,000 hospitalizations (359.7 for the ICU and 8.3 for non-ICUs) (Table 2).

Moreover, in the ICU, infections caused by *A. baumannii*, *K. pneumoniae*, *Staphylococcus aureus* and *Enterococcus faecalis* were predominant. *A. baumannii* was the most common cause of PNAs, *E. faecalis* in UTIs, and *E. faecium* and *K. pneumoniae* in BSIs (Table 5). *E. coli* and *K. pneumoniae* were dominant in UTIs. *A. baumannii* was a microorganism characterized by a high level of drug resistance. In our study, it showed the greatest sensitivity to colistin – only 3 strains of *A baumannii* were resistant to colistin with MIC > 4ug/mL – and to a lesser extent to aminoglycosides, independent of where the isolate was collected (Table 6). *E. coli* showed the greatest sensitivity to carbapenems, fosfomycin and nitroxoline.

**Table 5:** Most frequent etiologies of HAIs in COVID-19 patients at UHK

Etiological factors	ICU N (%)										Non-ICU N (%)					
	PNA		UTI		BSI		Others		**Total**		UTI		Others		**Total**	
**Gram-positive bacteria**																
* Staphylococcus aureus*	22	19.0	1	1.2	6	12.0	2	20.0	**31**	**11.8**	1	1.9	4	25.0	**5**	**7.2**
Coagulase Negative Staphylococci	0	0.0	0	0.0	5	10.0	0	0.0	**5**	**1.9**	0	0.0	1	6.3	**1**	**1.4**
* Streptococcus spp.*	3	2.6	1	1.2	3	6.0	0	0.0	**7**	**2.7**	0	0.0	2	12.5	**2**	**2.9**
* Enterococcus faecalis*	0	0.0	22	25.6	7	14.0	1	10.0	**30**	**11.5**	7	13.2	0	0.0	**7**	**10.1**
* Enterococcus faecium*	0	0.0	10	11.6	8	16.0	1	10.0	**19**	**7.3**	4	7.5	0	0.0	**4**	**5.8**
**Gram-negative bacteria**																
* Enterobacteriaceae*																
* Escherichia coli*	7	6.0	17	19.8	1	2.0	1	10.0	**26**	**9.9**	19	35.8	0	0.0	**19**	**27.5**
* Klebsiella pneumoniae*	16	13.8	5	5.8	8	16.0	2	20.0	**31**	**11.8**	11	20.8	1	6.3	**12**	**17.4**
* Enterobacter cloacae*	8	6.9	2	2.3	2	4.0	1	10.0	**13**	**5.0**	1	1.9	0	0.0	**1**	**1.4**
Others	11	9.5	9	10.5	2	4.0	1	10.0	**23**	**8.8**	4	7.5	0	0.0	**4**	**5.8**
Nonfermenting bacilli																
* Acinetobacter baumannii*	39	33.6	12	14.0	5	10.0	0	0.0	**56**	**21.4**	2	3.8	5	31.3	**7**	**10.1**
* Pseudomonas aeruginosa*	4	3.4	7	8.1	3	6.0	1	10,0	**15**	**5.7**	1	1.9	1	6.3	**2**	**2.9**
* Nonfermenting bacilli, others*	4	3.4	0	0.0	0	0.0	0	0.0	**4**	**1.5**	0	0.0	1	6.3	**1**	**1.4**
Other Gram-negative bacteria	2	1.7	0	0.0	0	0.0	0	0.0	**2**	**0.8**	3	5.7	1	6.3	**4**	**5.8**
**Total**	**116**	**100.0**	**86**	**100.0**	**50**	**100.0**	**10**	**100.0**	**262**	**100.0**	**53**	**100.0**	**16**	**100.0**	**69**	**100.0**

Legend: BSI: bloodstream infection; HAI: healthcare-associated infections; ICU: intensive care unit; PNA: pneumonia; UTI: urinary tract infection; UHK: University Hospital in Kraków

**Table 6:** Cumulative antibiogram based on the most frequent isolates in PNA and UTI cases

Antibiotics	PNA N = 118			UTI N = 136				
	* A. baumannii* n = 42	* S. aureus* n = 25	* K. pneumoniae* n = 18	*E. faecalis* n = 30	*E. coli* n = 36	* A. baumannii* n = 14	* K. pneumoniae* n = 14	
**Beta-lactam antibacterials: penicillins with extended spectrum, beta-lactamase resistant penicillins, combinations of penicillins incl. beta-lactamase inhibitors**								
Piperacillin	n/a	87%	0%	n/a	47%	n/a	7%	
Ampicillin	n/a	87%	n/a	100%	47%	n/a	n/a	
Ampicillin-sulbactam	n/a	87%	72%	100%	83%	n/a	43%	
Amoxicillin-clavulanic acid	n/a	87%	44%	100%	83%	n/a	57%	
Piperacillin-tazobactam	n/a	87%	50%	100%	92%	n/a	57%	
**Other beta-lactam antibacterials: second/third generation cephalosporins, carbapenems**								
Imipenem	12%	87%	94%	0%	100%	0,0	100%	
Meropenem	14%	87%	94%	n/a	100%	7%	100%	
Cefuroxime	n/a	87%	6%	n/a	86%	n/a	50%	
Cefotaxime	n/a	87%	61%	n/a	86%	n/a	57%	
Ceftazidime	n/a	87%	n/a	n/a	86%	n/a	n/a	
Cefepime	n/a	87%	61%	n/a	92%	n/a	64%	
Ceftazidime-avibactam	n/a	87%	100%	n/a	n/a	n/a	100%	
**Aminoglycoside antibacterials**								
Amikacin	26%	96%	83%	n/a	92%	21%	86%	
Gentamicin	55%	100%	78%	20%	50%	71%	71%	
Tobramycin	31%	96%	67%	n/a	86%	14%	57%	
**Quinolone antibacterials**								
Ciprofloxacin	2%	0%	61%	n/a	78%	0%	57%	
Levofloxacin	2%	0%	61%	n/a	78%	0%	50%	
Moxifloxacin	n/a	80%	n/a	n/a	n/a	n/a	n/a	
**Other antibacterials**								
Colistin	95%	n/a	n/a	n/a	n/a	93%	n/a	
Trimethoprim-sulfamethoxazole	19%	100%	78%	n/a	78%	29%	57%	
Fosfomycin	n/a	n/a	n/a	n/a	100%	n/a	n/a	
Nitrofurantoin	n/a	n/a	n/a	100%	89%	n/a	n/a	
Nitroxoline	n/a	n/a	n/a	n/a	100%	n/a	n/a	
Rifampicin	n/a	100%	n/a	n/a	n/a	n/a	n/a	
Teicoplanin	n/a	100%	n/a	100%	n/a	n/a	n/a	
Vancomycin	n/a	100%	n/a	100%	n/a	n/a	n/a	
Tetracycline	n/a	96%	n/a	n/a	n/a	n/a	n/a	
Tigecycline	n/a	100%	n/a	n/a	n/a	n/a	n/a	
Clindamycin	n/a	76%	n/a	n/a	n/a	n/a	n/a	
Erythromycin	n/a	76%	n/a	n/a	n/a	n/a	n/a	
Linezolid	n/a	100%	n/a	100%	n/a	n/a	n/a	

Legend: n/a: not available; PNA: pneumonia; UTI: urinary tract infection; A. baumannii, Acinetobacter baumannii; E. faecalis, Enterococcus faecalis; E.coli, Escherichia coli; K. pneumoniae, Klebsiella pneumoniae; S. aureus, Staphylococcus aureus

PFGE typing demonstrated that almost all the 43 *K. pneumoniae* isolates had unique pulsotypes and the other XDR strains also varied widely from each other. Yet there were a few identical pulsotypes, two of which were isolated from PNA and the other three were isolated from PNA and BSI (supplementary material).

## Discussion

The incidence of different types of HAIs observed in this study was high. It was 13.8% in the ICU and 5.7% in non-ICU settings (Table 2) while 2017 European data – before the COVID-19 pandemic – showed only 8.3% of ICU-patients presented with at least one HAI [15]. The COVID-19 pandemic’s strain on the health care system and the significant risk of HAI is further exemplified by Conway et al [16] in a multicenter, international, observational study where the bacterial HAI incidence in COVID-19 patients was 54%.^19^ The dominance of PNA is also in line with expectations; in our study it accounted for 46% of all HAIs in the ICU, similar to Conway et al. where it was 44%.^16^

The burden of XDR organisms from 2020 to 2021 at our hospital was substantial. Unfortunately, detailed data from other areas of Poland or from this region of Europe are not known.

Our result, though, is supported by data at a national level in Poland by the World Health Organizations (WHO) and European Centre for Disease Prevention and Control (ECDC) [17]. In these data there were rises in carbapenem resistance in Poland from 2016 to 2020 in *K. pneumoniae* isolates (8.2%) (based on invasive isolates only). This is in contrast with Western Europe where the overall prevalence of carbapenem resistance is much lower [18]. In addition, there can be substantial geographical and temporal variations in AMR, even at a sub-national level. Unfortunately, ECDC data are based on AMR data from invasive isolates reported to the European Antimicrobial Resistance Surveillance Network (EARS-Net), which means that data on non-invasive infections – especially pneumonia – are lacking. Taking this into account, the situation is likely more serious than the EARS-Net results indicate [19].

The most common hospital acquired infections in our study were pneumonias and urinary tract infections which is consistent with other data for European countries [20]. In the study population, more pneumonia cases were found in the ICU – accounting for almost half of all HAIs cases – than in non-ICU settings, reflecting the overall increased severity of these infections requiring higher levels of care. In our study, there were 24.8 carbapenem resistance cases per 10,000 hospitalizations which is much higher than other parts of the world such as the 3.36–3.79 cases per 10,000 hospitalizations seen nationally in the United States before COVID-19 pandemic [21]. Of the *A. baumannii* isolates tested in Poland in 2020, 78.2% were resistant to carbapenems and 88.3% to fluoroquinolones [20]. These numbers in Poland have been on the rise in the last 5 years. In our study, 85.7% of *A. baumannii* isolates were resistant to carbapenems and 98% to fluoroquinolones [20]. This was much higher than other parts of the world such as the United States where cases of carbapenem resistant *A. baumannii* have fallen from 2012 to 2017 (from 3.33 to 2.47 cases per 10,000) [21].

These types of findings have profound implications for choosing empiric antibiotics. For example, at this hospital, concern for an MDR organism would be high for a patient being admitted to the ICU with an infection given that 25% of such cases had MDR organisms. Almost 1/3 of pneumonia cases were related to *A. baumannii* and based on Table 6, pneumonia with *A. baumannii* was likely highly resistant. Empiric therapy to ensure appropriate antibiotic coverage would include colistin. This is concerning given that colistin is associated with significant toxicities [22]. Gathering such local susceptibility data for front line workers to choose appropriate antibiotics is essential for improving patient outcomes, especially in clinical environments with high rates of antimicrobial resistance [23]. These data can be further applied to different hospital locations (i.e., ICU vs. non-ICU) as these locations have differing antibiotic use and thus different antibiotic susceptibilities, making a general hospital-wide cumulative antibiogram less useful. An example of this is a study that developed a cumulative antibiogram for ICU patients with infections from a respiratory source and developed an optimized empiric regimen for patients being admitted to the ICU to help streamline decision-making for specific infectious processes [24]. The difficulty in treating these resistant organisms likely contributes to the excess deaths seen worldwide from antimicrobial resistance [1].

In the decade before the COVID-19 pandemic, Poland was already higher than average in antibiotic use in Europe with associated high rates of antimicrobial resistance [25]. Studies have shown that antibiotic use in patients with COVID-19 far exceeds the expected prevalence of bacterial superinfection [26]. This combination of factors puts Poland at risk for an increase in AMR prevalence and future studies should be pursued evaluating changes to Poland’s AMR epidemiology after the effects of COVID-19 on the health system. The increase in antibiotic use during COVID-19 is likely due to the difficulty of differentiating COVID-19 pneumonia from one with COVID-19 plus a bacterial superinfection, as the virus can cause tissue and immune disruptions that leads to colonizing bacteria turning pathogenic. This leads to empiric antibiotic coverage in many of these critically ill patients [27]. Studies have shown that proper stewardship of antibiotics (using the “right antibiotic, at the right time, right dose, right duration”) helps improve antimicrobial resistance rates [28, 29]. As an example of this, the United States saw a fall in most cases of antimicrobial resistance from 2012 to 2017 and during this time the number of US hospitals meeting CDC’s antimicrobial stewardship principles had doubled [30, 31].

Given the difficulty in treating XDR organisms with currently available antibiotics, prevention efforts that curb the spread of XDR organisms in hospital settings are key. These include measures such as barrier/contact precautions, patient cohorting and active surveillance. Spread of XDR organisms has been reported in hospitals that had more than one patient in a single room due to space constraints during the COVID-19 pandemic’s influx of hospitalized patients [32]. Of note, the isolates collected in this study are mostly genetically distinct from each other, suggesting that intrahospital spread of the same clonal organism is less likely. Specifically, PFGE did not confirm the horizontal spread of the strains of *K. pneumoniae* –almost all had unique pulsotypes, including the XDR strains. Our previous research on antimicrobial resistant strains of Enterobacterales derived from neonatal intensive care units [20] pointed to a major problem with the horizontal spread of epidemic *K. pneumoniae* clones, hence, the interest in evaluating whether there are strains with high genetic similarity among the currently collected XDR and non-XDR *K. pneumoniae* strains. However, in this sample set, the diversity of *K*. *pneumoniae* strains by PFGE was very high — only 5 strains were very similar. Therefore, this indicates that the cases of infection in this study were not associated with horizontal transfer.

Another major approach to addressing the high level of XDR organisms in our study is a robust antimicrobial stewardship program. For a successful program, a cultural shift is often necessary, especially in Poland. Several surveys were done involving the Polish public from 2009 to 2011 [33]. The results showed that 40% of respondents (regardless of the sex, age, education, and profession) expected to receive antibiotics for viral infections. While most of the public knew that antibiotics killed bacteria, many believed they also worked against viruses. Reassuringly, almost half of respondents had a change in attitude towards antibiotics after educational campaigns. Given that almost all these respondents received antibiotics as prescriptions from providers, an assessment of provider attitudes toward antibiotic use is essential. A study in 2017 analyzed physician attitudes toward antibiotic prescriptions and antimicrobial resistance in Poland [33]. Almost all physicians (regardless of the workplace, inpatient or outpatient care) surveyed believed Poles overused antibiotics. Most physicians knew about national recommendations guiding antibiotic use against certain infections, but many did not use microbiological/epidemiologic factors in determining antibiotic use. These surveys showed that there is an understanding from providers about the need to optimize antibiotic prescribing and there are opportunities for educating the Polish public on antibiotic use expectations. This can be channeled into programs that combine high quality cumulative antibiograms that inform empiric regimens with antimicrobial stewardship to combat antimicrobial resistance while improving patient outcomes. At UHK, these needs were revealed during the COVID-19 pandemic in the form of high MDR prevalence, and the described infection control and antibiotic stewardship practices will be critical to address these resistance issues.

## Limitations

Although this study provides valuable data regarding the prevalence and degree of resistance of MDR/XDR organisms during the COVID-19 pandemic, there are some limitations to our study. Unfortunately, it was not possible to compare the incidence of HAIs or XDRs in COVID-19 patients with non-COVID-19 patients within UHK or to pre-pandemic levels because UHK did not conduct active or passive microbial surveillance of those comparators. Instead, we include data in the discussion from other countries as a general method of comparison. Secondly the antibiotic susceptibility tests were done by the hospital diagnostic laboratory, which conducted tests exclusively for clinical purposes.

## Conclusions

XDR prevalence in this Polish hospital was high during the COVID-19 pandemic and higher compared to data from other geographic areas such as Western Europe and the United States. The highest prevalence of XDR was associated with pneumonia and with *A. baumannii*. hence pneumonia seems to be the most important problem of modern hospital microbiology in Poland due to the limited antibiotic options for empiric therapy. Possible ways to address the high prevalence of XDR organisms include implementation of improved antimicrobial stewardship with cumulative antibiograms to create local treatment guidelines and infection control practices. On the other hand, epidemics are a recurring phenomenon. The recent COVID-19 pandemic should become a source of knowledge about the problems faced from a new viral infection spreading quickly through a population;, the issue was not only the lack of targeted antiviral treatment, but also the general functioning of hospitals and the high prevalence of multidrug-resistant bacteria.

### Electronic supplementary material

Below is the link to the electronic supplementary material.

Supplementary Material 1

## Data Availability

The datasets analyzed during the current study are available from Jadwiga Wojkowska-Mach (e-mail: jadwiga.wojkowska-mach@uj.edu.pl) upon reasonable request.
