# Supplementary material for: Secondary bacterial infections & extensively drug-resistant bacteria among COVID-19 hospitalized patients at the University Hospital in Kraków
Source: Ann Clin Microbiol Antimicrob. 2023 Aug 24;22:77. doi: 10.1186/s12941-023-00625-8 (PMC10463524; doi:10.1186/s12941-023-00625-8)
Supplement: Supplementary file 1 — Supplementary Material 1 [file 12941_2023_625_MOESM1_ESM.pdf]

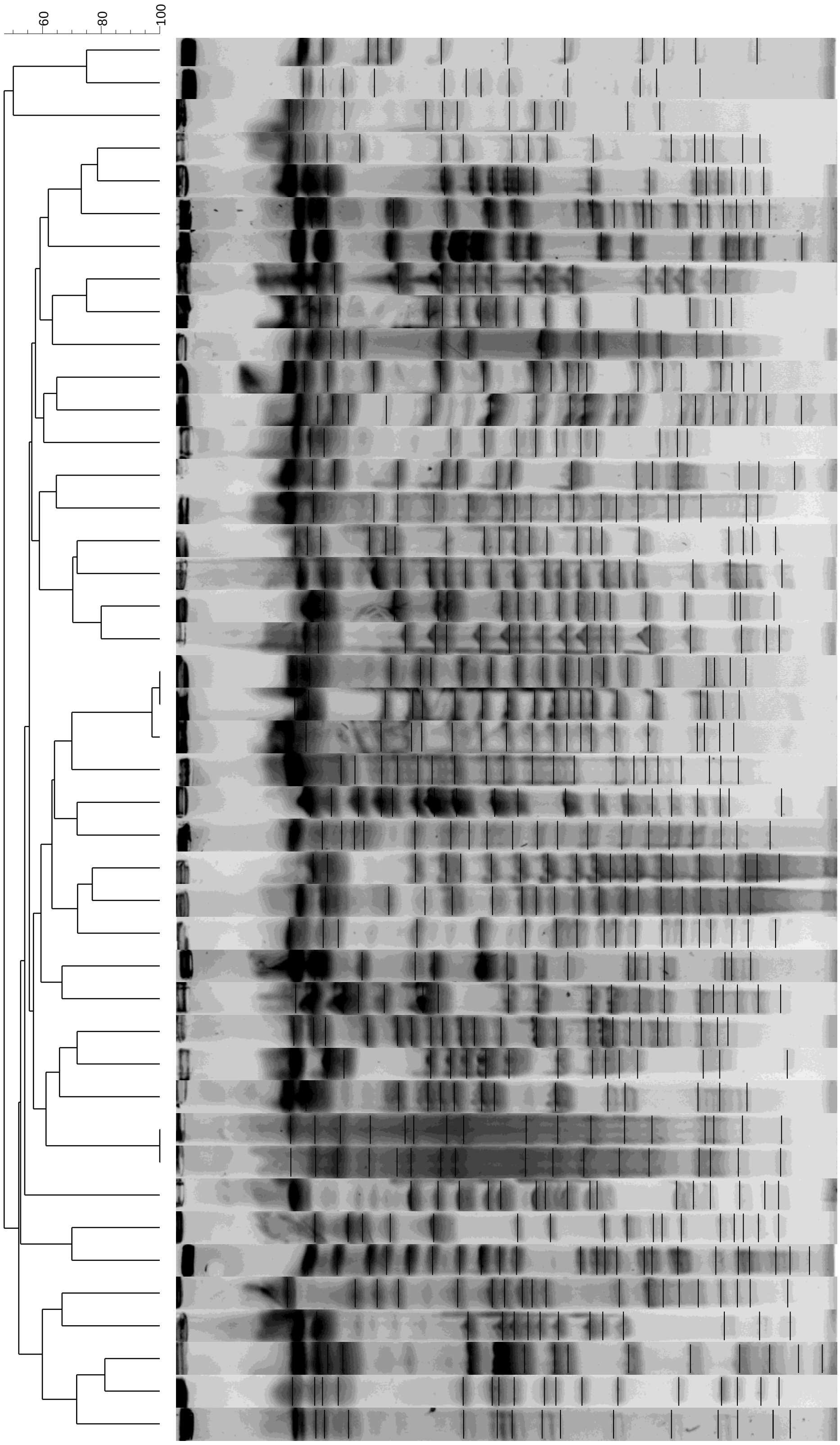

Strain number	Type of infection	XDR
SU13	PNA	
SU15	BSI	
SU313	PNA	yes
SU323	PNA	
SU360	BSI	
SU77	OTHER	yes
SU50	PNA	
SU188	UTI	
SU307	UTI	
SU345	BSI	
SU267	PNA	
SU45	UTI	yes
SU526	UTI	
SU417	PNA	
SU509	PNA	yes
SU161	UTI	
SU478	PNA	
SU166	UTI	yes
SU495	UTI	yes
SU269	BSI	
SU283	PNA	
SU292	PNA	
SU573	PNA	
SU380	OTHER	
SU58	UTI	
SU418	BSI	
SU448	PNA	yes
SU147	BSI	
SU120	UTI	
SU274	BSI	
SU390	UTI	
SU512	OTHER	
SU402	BSI	
SU468	PNA	
SU477	PNA	
SU503	UTI	
SU215	PNA	
SU32	PNA	
SU211	PNA	
SU452	UTI	
SU142	UTI	yes
SU150	UTI	yes
SU92	UTI	
